# Numerical Solution to Generalized Burgers'-Fisher Equation Using Exp-Function Method Hybridized with Heuristic Computation

**DOI:** 10.1371/journal.pone.0121728

**Published:** 2015-03-26

**Authors:** Suheel Abdullah Malik, Ijaz Mansoor Qureshi, Muhammad Amir, Aqdas Naveed Malik, Ihsanul Haq

**Affiliations:** 1 Department of Electronic Engineering, Faculty of Engineering and Technology, International Islamic University, Islamabad, Pakistan; 2 Department of Electrical Engineering, Air University, Islamabad, Pakistan; UCLA, UNITED STATES

## Abstract

In this paper, a new heuristic scheme for the approximate solution of the generalized Burgers'-Fisher equation is proposed. The scheme is based on the hybridization of Exp-function method with nature inspired algorithm. The given nonlinear partial differential equation (NPDE) through substitution is converted into a nonlinear ordinary differential equation (NODE). The travelling wave solution is approximated by the Exp-function method with unknown parameters. The unknown parameters are estimated by transforming the NODE into an equivalent global error minimization problem by using a fitness function. The popular genetic algorithm (GA) is used to solve the minimization problem, and to achieve the unknown parameters. The proposed scheme is successfully implemented to solve the generalized Burgers'-Fisher equation. The comparison of numerical results with the exact solutions, and the solutions obtained using some traditional methods, including adomian decomposition method (ADM), homotopy perturbation method (HPM), and optimal homotopy asymptotic method (OHAM), show that the suggested scheme is fairly accurate and viable for solving such problems.

## Introduction

Most physical phenomena arising in various fields of engineering and science are modeled by nonlinear partial differential equations (NPDEs). The investigation of solutions to NPDEs has attracted much attention due to their potential applications and many numerical schemes have been proposed, see for example [1–4]. The generalized Burgers′-Fisher equation is one of the important NPDE which appears in various applications, such as fluid dynamics, shock wave formation, turbulence, heat conduction, traffic flow, gas dynamics, sound waves in viscous medium, and some other fields of applied science [5–10].

The generalized Burgers′-Fisher equation is of the form [10–12]

$$u_{t}+\alpha u^{\delta }u_{x}-u_{xx}=\beta u\left(1-u^{\delta }\right)\;\forall x\in \left(0,1\right),t\geq 0$$ (1)

subject to the following initial condition

$$u\left(x,0\right)={\left(\frac{1}{2}+\frac{1}{2}\tanh\left(\frac{-\alpha \delta }{2\left(\delta +1\right)}x\right)\right)}^{\frac{1}{\delta }}$$ (2)

The exact solution is given by [10–12]

$$u_{exact}\left(x,t\right)={\left(\frac{1}{2}+\frac{1}{2}\tanh\left(\frac{-\alpha \delta }{2\left(\delta +1\right)}\left(x-\left(\frac{\alpha }{\delta +1}+\frac{\beta \left(\delta +1\right)}{\alpha }\right)t\right)\right)\right)}^{\frac{1}{\delta }}$$ (3)

Many researchers have investigated the analytical and numerical solutions of the generalized Burgers′-Fisher Equation (1) by using several different methods [8–17]. For example, Ismail et al. [11] used adomian decomposition method (ADM), Rashidi et al. [12] employed homotopy perturbation method (HPM), Nawaz et al. [10]applied optimal homotopy asymptotic method (OHAM),for obtaining approximate solutions of the generalized Burgers′-Fisher Equation (1). Very recently Mittal and Tripathi [8] employed modified cubic B-spline functions for the numerical solution of generalized Burgers′-Fisherand Burgers′-Huxley equations. Khattak [13] used collocation based radial base functions method (CBRBF) for numerical solution of the generalized Burgers′-Fisher equation. Javidi [14] used modified pseudospectral method for generalized Burgers′-Fisher equation.

The Exp-function method was introduced recently by He and Wu [18] to obtain the generalized solitonary solutions and periodic solutions of nonlinear wave equations. The method has attracted much attention due to its simple and straightforward implementation and many authors used it [19–24]. Among many authors, Xu and Xian [19] used Exp-function method for obtaining the solitary wave solutions for generalized Burgers′-Fisher equation. Özişand Köroğlu[20] used Exp-function method for obtaining travelling wave solutions of the Fisher equation. Chun[21] used Exp-function method for solving Burgers′-Huxley equation.

In recent years, many authors have used heuristic computation based techniques for solving variety of differential equations [25–35]. Very recently Malik et al. [25,26]used nature inspired computation based approach for solving systems of nonlinear ordinary differential equations (NODEs), including biochemical reaction model [25], and boundary value problems arising in physiology [26]. Khan et al.[27] used evolutionary computation (EC) based artificial neural network (ANN)method for solving van der pol oscillator equation. Arqab et al. [28] used genetic algorithm (GA) based method for solving linear and nonlinear ODEs. Caetano et al. [29] used the ANN based method for solving NODEs arising in atomic and molecular physics.

The aim of this work is to obtain the approximate solution of the generalized Burgers′-Fisher equation using a novel scheme. The scheme is based on the elegant hybrid approach of Exp-function method and evolutionary algorithm (EA). In the proposed scheme the Exp-function method is used to express the approximate wave solution with unknown parameters. The given NPDE is converted into a global error minimization problem using a fitness function with unknown parameters. Genetic algorithm (GA), one of the renowned evolutionary algorithms is adopted for solving the minimization problem and to achieve the unknown parameters.

To the best of our knowledge nobody as yet has tackled with the generalized Burgers′-Fisher equation with the scheme presented in this work. The proposed scheme is simple and straightforward to implement and also gives the approximate solution at any value of choice in the solution domain. The efficiency and reliability of the proposed scheme is illustrated by solving generalized Burgers′-Fisher and Burgers′ equations successfully.

## Materials and Methods

In this section, stochastic global search algorithm GA is introduced, the basic idea of Exp-function method is given, and description of the proposed scheme is provided.

### Genetic algorithm (GA)

Genetic algorithm (GA) is one of the well-known evolutionary algorithms (EAs) that find the optimal solution of a problem from a randomly generated population of individuals called chromosome. Each individual within a population is regarded as a possible solution to the problem. The individuals within a population are evaluated using a fitness function that is specific to the problem at hand. The algorithm evolves population iteratively by means of three primary operations: selection, crossover, and mutation to reach the optimal solution [36].

The procedural steps of GA are given in algorithm 1, while the parameters settings of the algorithm used in this work are given in Table 1.

**Table 1:** Parameter settings and values for GA.

Parameter Name	Setting/Value	
	Example 1	Example 2
Population size	[310 310]	[310 310]
Chromosome size	12	12
Scaling function	Rank	Proportional
Selection function	Stochastic uniform	Stochastic uniform
Mutation function	Adaptive feasible	Adaptive feasible
Crossover function	Heuristic	Heuristic
Crossover fraction	0.8	0.9
No. of generations	1000	1000
Function tolerance	1e-18	1e-18
Bounds	-10, +10	-10, +10

#### Algorithm 1

**Step 1**: (Population Initialization)

A population of N individuals or chromosomes (**C**_1_, **C**_2_, ….,**C**_N_) each of length M is generated using random number generator. The length of each chromosome represents the number of unknown parameters.

**Step 2**: (Fitness Evaluation)

A problem exclusive fitness function is used to compute the fitness of each chromosome.

**Step 3**: (Selection and Reproduction)

The chromosomes from the current population are chosen on the basis of their fitness value which acts as parents for new generation. These parents produce children (offsprings) with a probability to their fitness through crossover operation.

**Step 4**: Mutation

Mutation operation introduces random alterations in the genes to maintain the genetic diversity to find a good solution.

**Step 5**: (Stoppage Criteria)

The algorithm terminates if the maximum number of cycles has exceeded or a predefined fitness value is achieved. Else go to step 3.

### Overview of Exp-function method

Consider a nonlinear partial differential equation (NPDE) given in the following form

$$N\left(u,u_{x},u_{t},u_{xx},u_{tt},u_{xt}\right)=0$$ (4)

Using a transformation, *u(x*,*t) = u(η)* with *η* defined as follows

η=kx+ωt (5)

Equation (4) is converted into a following ODE

$$P\left(u,ku',\omega u',k^{2}u'',....\right)=0$$ (6)

where *k* and ω are unknown constants, and prime denotes derivation with respect to *η*.

According to Exp-function method [18], the solution of (6) is expressed in the following form

$$u\left(\eta \right)=\frac{\sum_{n=-c}^{d}a_{n}\exp\left(n\eta \right)}{\sum_{m=-p}^{q}b_{m}\exp\left(m\eta \right)}=\frac{a_{-c}\exp\left(-c\eta \right)+........+a_{d}\exp\left(d\eta \right)}{b_{-p}\exp\left(-p\eta \right)+........+b_{q}\exp\left(q\eta \right)}$$ (7)

where *c*, *d*, *p*, and *q* are unknown positive integers, *a*_*n*_ and *b*_*m*_ are unknown constants.

The values of c and p are determined by balancing the linear term of highest order in (6) with the highest order nonlinear term, which gives *p = c*[18, 37]. Similarly the values of d and q are determined by balancing the lowest order of linear and nonlinear terms in (6), which yields *q = d*[18, 37]. Once *c*, *d*, *p*, *q* are determined their values are freely chosen [18]. Next the unknown constants *a*_*n*_ and *b*_*m*_ are determined by substituting (7) into (6) and equating the coefficients of exp(*nη*) to zero, which results into a set of algebraic equations with unknown constants. The systems of algebraic equations are solved using some software package like Matlab, Maple or Mathematica for determining the unknown constants *a*_*n*_ and *b*_*m*_, Consequently the solution of NPDE (4) is obtained.

### Description of the proposed scheme

We consider the NPDE given by (4) subject to the following initial condition

u(x,0)=f(x) (8)

Apply the transformation variable *η = kx + ωt* to (4) yields NODE given by (6). We assume that the approximate solution of (6) is expressed in the following form in view of the Exp-function method [18].

$$\overset{⌢}{u}\left(\eta \right)=\frac{a_{-c}\exp\left(-c\eta \right)+........+a_{d}\exp\left(d\eta \right)}{b_{-c}\exp\left(-c\eta \right)+........+b_{d}\exp\left(d\eta \right)}$$ (9)

As mentioned above the values of *c* and *d* can be feely chosen, therefore we accordingly set their values. The rest of the unknown parameters existing in (9) including (*a*_-*c*_,*…*,*a*_*d*_;*b*_-*c*_,*…*,*b*_*d*_;*k*,*ω*) need to be found to obtain the approximate solution of (6). To determine the values of these unknown parameters, the transformed NODE (6) along with the initial condition (8) is converted into an equivalent global error minimization problem by developing a trial solution using a fitness function (FF). The fitness function (FF) consists of the sum of two parts. The first part represents the mean of sum of the square errors associated with the transformed NODE (6), and the second part represents the mean of sum of the square errors associated with the initial condition (8), which are given respectively as follows

ε1=1N×S∑i=1N∑j=1S(P(u⌢(kxj+ωti),ku⌢'(kxj+ωti),k2u⌢''(kxj+ωti),...))2 (10)

$$\varepsilon_{2}=\frac{1}{S}\sum_{j=1}^{S}{\left(\overset{⌢}{u}\left(x_{j},0\right)-f\left(x_{j}\right)\right)}^{2}$$ (11)

where *N* and *S* are the total number of steps taken in the solution domain of *x* and *t*, and $\overset{⌢}{u}$, u⌢', u⌢'' are given by (9) and its derivates respectively.

The FF which is denoted as *ε*_*j*_ is accordingly formulated as follows

$$\varepsilon_{j}=\varepsilon_{1}+\varepsilon_{2}$$ (12)

where *j* is the generation index.

The error minimization problem given by (12) is solved using the application of evolutionary algorithm, such as GA, to find the optimal values of unknown parameters (*a*_-*c*_,*…*.*a*_*d*_;*b*_-*c*_,*…*,*b*_*d*_;*k*,*ω*). Once the values of the unknown parameters are achieved, they are used in (9), which consequently provides the approximate numerical solution of the given NPDE.

### Numerical approximation of generalized Burgers′-Fisher equation

To solve the generalized Burgers′-Fisher Equation (1) using the proposed scheme, we first apply the transformation variable *η = kx + ωt* which yield the following NODE

$$\omega u'+\alpha u^{\delta }ku'-k^{2}u''=\beta u\left(1-u^{\delta }\right)$$ (13)

Assume the approximate solution of (13) is given by (9) in the view of the Exp-function method [18]. To determine the unknown parameters (*a*_-*c*_,*…*,*a*_*d*_;*b*_-*c*_,*…*,*b*_*d*_;*k*,*ω*) in (9) for obtaining the approximate solution, the FF is formulated as follows (14)—(16)

$$\varepsilon_{1}=\frac{1}{\left(N\times S\right)}\sum_{i=1}^{N}\sum_{j=1}^{S}{\left(\begin{array}{l} \omega u'\left(kx_{j}+\omega t_{i}\right)+\alpha u^{\delta }\left(kx_{j}+\omega t_{i}\right)ku'\left(kx_{j}+\omega t_{i}\right)\\ -k^{2}u''\left(kx_{j}+\omega t_{i}\right)-\beta u\left(kx_{j}+\omega t_{i}\right)\left(1-u^{\delta }\left(kx_{j}+\omega t_{i}\right)\right) \end{array}\right)}^{2}$$ (14)

$$\varepsilon_{2}=\frac{1}{S}\sum_{j=1}^{S}{\left(u\left(x_{j},0\right)-{\left(\frac{1}{2}+\frac{1}{2}\tanh\left(-\frac{\alpha \delta }{2\left(\delta +1\right)}x_{j}\right)\right)}^{\frac{1}{\delta }}\right)}^{2}$$ (15)

$$\varepsilon_{j}=\varepsilon_{1}+\varepsilon_{2}$$ (16)

The FF given by (16) contains unknown parameters in the form of a chromosome for GA. The objective is to solve the global error minimization problem given by Equation (16) and to achieve the optimal chromosome which represents the values of unknown parameters. Consequently the approximate solution $\overset{⌢}{u}\left(\eta \right)$ of the generalized Burgers′-Fisher equation is obtained using the values of the unknown parameters in (9).

### Convergence of the Proposed Scheme

Let the exact solution be *g*(*η*). By Exp-function method we get the solution *u*(*η*) as follows

$$u\left(\eta \right)=\frac{a_{-c}\exp\left(-c\eta \right)+........+a_{d}\exp\left(d\eta \right)}{b_{-c}\exp\left(-c\eta \right)+........+b_{d}\exp\left(d\eta \right)}$$ (17)

This is a continuous function on a compact set. We apply Stone-Weierstrass theorem to prove that for any given *g*(*η*) on U and arbitrary *ε* > 0, there exists a system like *u*(*η*) as given above such that

|u(η)−g(η)|ηϵUsup<ε (18)

That is *u*(*η*)can be a universal approximator. For this three conditions given in Stone-Weierstrass theorem have to be satisfied.

Let Z be a set of real continuous functions like *u*(*η*) on a compact set U.

Condition 1: All these must be closed under addition, multiplication, and scalar multiplication.

As we can see that addition (*u*_1_(*η*) + *u*_2_(*η*)) will give same type of function. Similarly multiplication (*u*_1_(*η*) × *u*_2_(*η*)) will also give same type of function, which is real, continuous and on compact set of U. The same is true for scalar multiplication.

Condition 2: For every *η*_*1*_ *andη*_*2*_ *∈U*,*η*_1_ ≠ *η*_2_ there exists function *u*∈*Z* such that *u*(*η*_1_)≠*u*(*η*_2_)

Condition 3: *u*(*η*)≠0 for each *η*∈*U* As we can easily judge from the function that its numerator ≠0 for ∀*a_i_* > 0,*b_i_* > 0.

Thus with these three conditions satisfied, there exists for *g*(*η*) a function *u*(*η*) with arbitrary *ε* > 0 such that

|u(η)−g(η)|ηϵUsup<ε (19)

### Numerical Results and Discussion

In this section, we apply the proposed scheme to the Burgers′-Fisher equation to test and assess its performance and to demonstrate the efficacy of the proposed scheme. Further to prove the accuracy and reliability of the proposed scheme comparisons of the numerical results are made with the exact solutions and some traditional methods, including OHAM [10], ADM [11], HPM [12], and CBRBF [13]. For simulations, Matlab 7.6 has been utilized in this work.

#### Example 1

We consider the generalized Burgers′-Fisher equation transformed into NODE given by Equation (13) with the initial condition given by (2). The approximate solution is obtained in the domain *x* ∈ (0,1) and *t* ∈ (0,1) for different values of *α*,*β*,and *δ* as follows.

Case 1: *α* = *β* = 0.001,*δ* = 1

Case 2: *α* = *β* = 0.1,*δ* = 1

Case 3: *α* = *β* = 0.5,*δ* = 1

Case 4: *α* = *β* = 1,*δ* = 2

Case 5: *α* = 2,*β* = 5,*δ* = 3/2

As mentioned above that the values of *c* and *d* can be freely chosen, we set *p = c = 2* and *d = q = 2* in Equation (9), therefore we get the approximate solution in the form

$$\overset{⌢}{u}\left(\eta \right)=\frac{a_{-2}\exp\left(-2\eta \right)+a_{-1}\exp\left(-\eta \right)+a_{0}+a_{1}\exp\left(\eta \right)+a_{2}\exp\left(2\eta \right)}{b_{-2}\exp\left(-2\eta \right)+b_{-1}\exp\left(-\eta \right)+b_{0}+b_{1}\exp\left(\eta \right)+b_{2}\exp\left(2\eta \right)}$$ (20)

The unknown parameters (*a*_-2_,…,*a*_2_;*b*_-2_,…,*b*_2_;*k*,*ω*) in Equation (20) are achieved using the stochastic global search algorithm GA by formulating the fitness function given by Equations. (14)—(16). For instance the fitness function corresponding to case 2, with *N = 11* and *S = 11*is given by

$$\varepsilon_{1}=\frac{1}{121}\sum_{i=1}^{11}\sum_{j=1}^{11}{\left(\begin{array}{l} \omega u'\left(kx_{j}+\omega t_{i}\right)+\left(0.1\right)u\left(kx_{j}+\omega t_{i}\right)ku'\left(kx_{j}+\omega t_{i}\right)\\ -k^{2}u''\left(kx_{j}+\omega t_{i}\right)-\left(0.1\right)u\left(kx_{j}+\omega t_{i}\right)\left(1-u\left(kx_{j}+\omega t_{i}\right)\right) \end{array}\right)}^{2}$$ (21)

$$\varepsilon_{2}=\frac{1}{11}\sum_{j=1}^{11}{\left(u\left(x_{j},0\right)-\left(\frac{1}{2}+\frac{1}{2}\tanh\left(-\frac{0.1}{4}x_{j}\right)\right)\right)}^{2}$$ (22)

$$\varepsilon_{j}=\varepsilon_{1}+\varepsilon_{2}$$ (23)

Similarly we formulate fitness function corresponding to each case defined above. The parameter settings and values used for the implementation of GA are given in Table 1. The number of unknown parameters (*a*_-2_,…,*a*_2_;*b*_-2_,…,*b*_2_;*k*,*ω*) which need to be tailored is 12, therefore the size of chromosome is chosen as12. The values of these unknown adjustable parameters are restricted between -10 and +10. The global search algorithm GA is executed to achieve the minimum fitness, with the prescribed parameter settings and values given in Table 1.

The optimal chromosomes representing the values of unknown constants corresponding to the minimum fitness achieved by GA are provided in Table 2. Using the values of unknown constants from Table 2 in Equation (20), provides the approximate solution of the generalized Burgers′-Fisher equation at any value of *x* and *t* in the solution domain [0, 1].

**Table 2:** Optimal values of unknown constants acquired by GA for example 1.

Constant	Case 1	Case 2	Case 3	Case 4	Case 5
*a*_-*2*_	0.104865	4.865539	-0.454457	-3.276565	1.060624
*a*_-*1*_	0.003998	5.177595	4.749431	6.935221	1.163479
*a*_*0*_	0.440651	4.968585	3.375969	1.337205	0.805153
*a*_*1*_	0.170067	4.712250	-5.536516	9.076617	0.158400
*a*_*2*_	0.903084	0.134866	6.650495	-0.237617	-0.004869
*b*_-*2*_	1.150501	4.858927	7.013754	-3.081064	1.060638
*b*_-*1*_	0.107163	5.419075	2.313111	3.223797	1.160318
*b*_*0*_	1.346060	8.701317	2.590633	8.442465	0.855922
*b*_*1*_	0.816371	5.305503	-0.481886	9.103490	0.531301
*b*_*2*_	-0.174764	3.799747	6.134235	9.981056	1.441113
*k*	0.000148	-0.396473	0.035417	-0.222195	-0.396014
ω	-0.000297	1.321509	-0.072606	0.499930	2.785516

In Table 3 we have presented numerical solutions obtained by the proposed scheme for time *t* = 0.1 for case 1-case 4, also exact solutions are given for comparison. Table 4 shows absolute errors $\left|\left(u_{exact}-\overset{⌢}{u}\left(\eta \right)\right)\right|$ obtained by the proposed scheme at time *t = 0*.*1*for case 1—case 4. Further, in Table 5 a comparison of our numerical solutions is made with the exact solutions for various values of *x* and *t* for case 5.

**Table 3:** Numerical solutions of generalized Burgers′-Fisher equation by the proposed scheme for different values of *α*, *β*, *δ* and comparison with exact solutions for time *t* = 0.1.

*x*	*δ* = 1						*δ* = 2	
	α = β = 0.001		α = β = 0.1		α = β = 0.5		α = β = 1	
	Exact	Proposed	Exact	Proposed	Exact	Proposed	Exact	Proposed
	*u_exact_*	$\overset{⌢}{u}\left(\eta \right)$	*u_exact_*	$\overset{⌢}{u}\left(\eta \right)$	*u_exact_*	$\overset{⌢}{u}\left(\eta \right)$	*u_exact_*	$\overset{⌢}{u}\left(\eta \right)$
0.0	0.500025	0.500025	0.502562	0.502562	0.514059	0.514057	0.745203	0.745205
0.1	0.500013	0.500012	0.501312	0.501312	0.507812	0.507811	0.734037	0.734038
0.2	0.500000	0.500000	0.500062	0.500062	0.501562	0.501562	0.722639	0.722640
0.3	0.499988	0.499987	0.498813	0.498812	0.495313	0.495312	0.711024	0.711024
0.4	0.499975	0.499975	0.497563	0.497562	0.489064	0.489064	0.699207	0.699206
0.5	0.499963	0.499962	0.496313	0.496313	0.482819	0.482819	0.687205	0.687204
0.6	0.499950	0.499950	0.495063	0.495063	0.476580	0.476580	0.675035	0.675033
0.7	0.499938	0.499938	0.493813	0.493813	0.470347	0.470347	0.662715	0.662713
0.8	0.499925	0.499925	0.492563	0.492563	0.464124	0.464124	0.650264	0.650261
0.9	0.499913	0.499913	0.491313	0.491313	0.457912	0.457912	0.637701	0.637698
1.0	0.499900	0.499900	0.490064	0.490064	0.451713	0.451714	0.625046	0.625042

**Table 4:** The absolute errors for example 1 for different values of *α*, *β*, *δ* and for time *t* = 0.1.

*x*	*δ = 1*			*δ = 2*
	*α* = *β* = 0.001	*α* = *β* = 0.1	*α* = *β* = 0.5	*α* = *β* = 1
0.0	2.236E-08	8.009E-08	1.669E-06	1.396E-06
0.1	1.988E-08	7.001E-08	1.165E-06	8.651E-07
0.2	1.706E-08	5.985E-08	7.771E-07	3.266E-07
0.3	1.390E-08	4.967E-08	4.836E-07	2.146E-07
0.4	1.040E-08	3.954E-08	2.670E-07	7.568E-07
0.5	6.547E-09	2.954E-08	1.123E-07	1.303E-06
0.6	2.354E-09	1.972E-08	6.852E-09	1.859E-06
0.7	2.182E-09	1.018E-08	5.971E-08	2.436E-06
0.8	7.062E-09	9.795E-10	9.571E-08	3.047E-06
0.9	1.228E-08	7.780E-09	1.074E-07	3.704E-06
1.0	1.785E-08	1.601E-08	9.900E-08	4.418E-06

**Table 5:** Comparison of numerical solutions and absolute errors for *α* = 2, *β* = 5, *δ* = 3/2.

*x*	*t*	*u_exact_*	$\overset{⌢}{u}\left(\eta \right)$	Absolute errors
0.1	0.2	0.881815	0.881912	9.65E-05
	0.4	0.975295	0.975367	7.15E-05
	0.6	0.995333	0.995292	4.15E-05
	0.8	0.999137	0.999127	9.74E-06
	1	0.999841	0.999874	3.31E-05
0.5	0.2	0.824570	0.824537	3.29E-05
	0.4	0.960817	0.960883	6.64E-05
	0.6	0.992485	0.992451	3.47E-05
	0.8	0.998605	0.998579	2.69E-05
	1	0.999743	0.999767	2.43E-05
1	0.2	0.727552	0.727337	2.15E-04
	0.4	0.931343	0.931303	3.99E-05
	0.6	0.986412	0.986394	1.83E-05
	0.8	0.997463	0.997416	4.66E-05
	1	0.999532	0.999540	8.35E-06

Tables 6 and 7 show the comparison of numerical solutions and absolute errors obtained by the proposed scheme, with the exact solutions, and absolute errors obtained by OHAM [10] and ADM [11], for *α* = *β* = 0.001,*δ* = 1 and *α* = *β* = 0.001,*δ* = 2 respectively. Further, Table 8 shows comparison of numerical solutions from the proposed scheme with the exact solutions, and absolute errors obtained by HPM [12].

**Table 6:** Comparison of numerical solutions and absolute errors between the proposed scheme, OHAM[10] and ADM [11] for *α* = *β* = 0.001 and *δ* = 1.

		Exact	Proposed	Absolute errors		
*x*	*t*	*u_exact_*	$\overset{⌢}{u}\left(\eta \right)$	Proposed	ADM [11]	OHAM [10]
0.1	0.001	0.499988	0.499988	1.97E-08	1.94E-06	2.25E-08
	0.005	0.499989	0.499989	1.97E-08	9.69E-06	1.12E-07
	0.01	0.499990	0.499990	1.97E-08	1.94E-06	2.25E-07
0.5	0.001	0.499938	0.499938	3.58E-09	1.94E-06	4.58E-08
	0.005	0.499939	0.499939	3.71E-09	9.69E-06	2.29E-07
	0.01	0.499940	0.499940	3.88E-09	1.94E-06	4.58E-07
0.9	0.001	0.499888	0.499888	1.80E-08	1.94E-06	4.58E-08
	0.005	0.499889	0.499889	1.77E-08	9.69E-06	2.29E-07
	0.01	0.499890	0.499890	1.74E-08	1.94E-06	4.58E-07

**Table 7:** Comparison of numerical solutions and absolute errors between the proposed scheme, OHAM [10] and ADM [11] for α = β = 1 and *δ* = 2.

		Exact	Proposed	Absolute errors		
*x*	*t*	*u_exact_*	$\overset{⌢}{u}\left(\eta \right)$	Proposed	ADM [11]	OHAM [10]
0.1	0.0001	0.695266	0.695267	1.08E-06	2.80E-04	1.17E-05
	0.0005	0.695426	0.695427	1.08E-06	1.40E-03	5.87E-05
	0.001	0.695625	0.695626	1.08E-06	2.80E-03	1.17E-04
0.5	0.0001	0.646130	0.646129	1.14E-06	2.69E-04	5.33E-05
	0.0005	0.646297	0.646296	1.14E-06	1.34E-03	1.06E-05
	0.001	0.646506	0.646505	1.14E-06	2.69E-03	1.06E-05
0.9	0.0001	0.595310	0.595306	4.12E-06	2.55E-04	9.29E-06
	0.0005	0.595481	0.595477	4.12E-06	1.27E-03	4.64E-05
	0.001	0.595695	0.595691	4.12E-06	2.55E-03	9.29E-04

**Table 8:** Comparison of numerical solutions and absolute errors between the proposed scheme and HPM [12] for *δ* = 1 at different values of *α* and *β*.

		*α* = *β* = 0.1				*α* = *β* = 0.5			
		Exact	Proposed	Absolute errors		Exact	Proposed	Absolute errors	
*t*	*x*	*u_exact_*	$\overset{⌢}{u}\left(\eta \right)$	Exact	Proposed	*u_exact_*	$\overset{⌢}{u}\left(\eta \right)$	Exact	Proposed
0.1	0.2	0.500062	0.500062	5.98E-08	4.32E-08	0.501562	0.501562	7.77E-07	6.17E-08
	0.4	0.497563	0.497562	3.95E-08	1.08E-07	0.489064	0.489064	2.67E-07	1.60E-05
	0.6	0.495063	0.495063	1.97E-08	1.74E-07	0.476580	0.476580	6.85E-09	2.58E-05
	0.8	0.492563	0.492563	9.80E-10	2.40E-07	0.464124	0.464124	9.57E-08	3.54E-05
0.4	0.2	0.507749	0.507749	6.75E-08	3.85E-07	0.543639	0.543631	7.40E-06	7.87E-05
	0.4	0.505250	0.505250	4.89E-08	6.65E-07	0.531209	0.531205	4.69E-06	7.89E-05
	0.6	0.502750	0.502750	2.93E-08	1.71E-06	0.518741	0.518738	2.95E-06	2.36E-04
	0.8	0.500250	0.500250	9.08E-09	2.76E-06	0.506250	0.506248	1.87E-06	3.92E-04
0.8	0.2	0.517992	0.517992	5.09E-08	7.28E-06	0.598688	0.598635	5.22E-05	1.24E-03
	0.4	0.515495	0.515495	4.27E-08	3.08E-06	0.586618	0.586583	3.48E-05	6.22E-04
	0.6	0.512997	0.512997	3.09E-08	1.12E-06	0.574443	0.574419	2.32E-05	2.80E-06
	0.8	0.510498	0.510498	1.63E-08	5.32E-06	0.562177	0.562161	1.54E-05	6.28E-04

From the comparison of numerical solutions and absolute errors, the efficiency and reliability of the proposed scheme is quite evident. Moreover, it is observed from the findings that the proposed scheme is more accurate than traditional methods including OHAM [10], ADM [11], and HPM [12].

#### Example 2

With *β* = 0 and *α* = 1 Equation (1) is reduced to the generalized Burgers′ equation [11].

The approximate solution is obtained by the proposed scheme for three different values of *δ* = 1,2,3 in the domain *x* ∈(0,1) and *t* ∈(0,2)for *δ* = 1,2, and t ∈(0,5) for *δ = 3*.

We assume the solution is expressed by Exp-function method given by Equation (20). The fitness function is developed for each value of *δ* with *β* = 0 and *α* = 1. For example, the fitness function with *δ = 3* is given as follows

$$\begin{array}{c} \varepsilon_{j}=\frac{1}{121}\sum_{i=1}^{11}\sum_{j=1}^{11}{\left(\omega u'\left(kx_{j}+\omega t_{i}\right)+u^{3}k\left(kx_{j}+\omega t_{i}\right)u'\left(kx_{j}+\omega t_{i}\right)-k^{2}u''\left(kx_{j}+\omega t_{i}\right)\right)}^{2}+\frac{1}{11}\sum_{j=1}^{11}{\left(u\left(x_{j},0\right)-{\left(\frac{1}{2}+\frac{1}{2}\tanh\left(\frac{-3}{8}x_{j}\right)\right)}^{\frac{1}{3}}\right)}^{2} \end{array}$$ (24)

GA is used to solve the minimization problem such as given by Equation (24) and to obtain the optimal values of unknown constants in Equation (20). The parameter settings for the implementation of GA are given in Table 1.

The optimal values of unknown constants achieved by GA are given in Table 9 for each value of *δ* = 1, 2, 3. The approximate solutions of generalized Burgers′ equation are obtained consequently by using the values of unknown constants in Equation (20).

**Table 9:** Optimal values of unknown constants acquired by GA for example 2 for different values of δ.

Constant	*δ* = 1	*δ* = 2	*δ* = 3
*a*_-*2*_	-0.021250	-1.170684	9.750017
*a*_-*1*_	0.095133	6.455429	-0.688173
*a*_*0*_	4.337944	1.201869	5.579583
*a*_*1*_	3.836255	5.953727	0.674093
*a*_*2*_	2.793948	9.851368	-0.331174
*b*_-*2*_	4.851505	9.542842	9.393467
*b*_-*1*_	3.339690	7.284266	1.566725
*b*_*0*_	7.418916	-3.834267	-0.004362
*b*_*1*_	3.654091	9.419307	8.977442
*b*_*2*_	2.819921	9.113024	-1.054169
*k*	0.245463	0.179999	-0.231315
ω	-0.122734	-0.059993	0.057836

In Tables 10–13 we provide the comparison of numerical solutions obtained by the proposed scheme with the exact solutions, and the solutions obtained by ADM [11] and CBRBF [13]. The comparisons of numerical solutions and absolute errors reveals that the proposed scheme is quite competent with other methods including ADM and RBF used in [11,13] for solving the generalized Burgers′ equation. The comparison further reveals that the proposed scheme is capable to achieve the approximate solutions in the larger domain of time *t* with greater accuracy. Moreover, for *δ* = 3 more accurate results are obtained by the proposed scheme as compared to ADM [11] and CBRBF [13].

**Table 10:** Numerical solutions of generalized Burgers′ equation by the proposed scheme and comparison with exact solutions, ADM [11], and RBF [13] for β = 0, α = 1, and *δ* = 1.

		Exact	Proposed	ADM	CBRBF	Absolute errors		
*t*	*x*	*u_exact_*	$\overset{⌢}{u}\left(\eta \right)$	[11]	[13]	Proposed	ADM	CBRBF
0.5	0.1	0.518741	0.518740	0.518741	0.518739	1.14E-07	6.34E-08	2.00E-06
	0.5	0.468791	0.468791	0.468791	0.468790	1.13E-07	5.66E-08	1.00E-06
	0.9	0.419458	0.419459	0.419458	0.419449	1.56E-06	4.12E-08	9.00E-06
1.0	0.1	0.549834	0.549833	0.549832	0.549831	1.17E-06	2.02E-06	3.00E-06
	0.5	0.500000	0.499999	0.499998	0.499998	3.79E-08	1.84E-06	2.00E-06
	0.9	0.450166	0.450167	0.450165	0.450157	1.28E-06	1.37E-06	9.00E-06
2.0	0.1	0.610639	0.610638	0.610575	0.610635	8.44E-07	6.42E-05	4.00E-06
	0.5	0.562177	0.562176	0.562116	0.562175	1.16E-07	6.06E-05	2.00E-06
	0.9	0.512497	0.512498	0.512450	0.512488	9.72E-07	4.75E-05	9.00E-06

**Table 11:** Numerical solutions of generalized Burgers′ equation by the proposed scheme and comparison with exact solutions, ADM [11], and CBRBF [13] for *β* = 0, *α* = 1, and *δ* = 2.

		Exact	Proposed	ADM	CBRBF	Absolute errors		
*t*	*x*	*u_exact_*	$\overset{⌢}{u}\left(\eta \right)$	[11]	[13]	Proposed	ADM	CBRBF
0.5	0.1	0.714919	0.714918	0.714919	0.714920	7.43E-07	1.25E-08	1.00E-06
	0.5	0.666837	0.666836	0.666837	0.666839	1.16E-06	1.49E-08	2.00E-06
	0.9	0.616567	0.616565	0.616567	0.616567	2.38E-06	1.39E-08	-
1.0	0.1	0.734037	0.734034	0.734037	0.734037	2.94E-06	1.25E-08	-
	0.5	0.687205	0.687202	0.687205	0.687206	3.22E-06	4.75E-07	1.00E-06
	0.9	0.637701	0.637697	0.637701	0.637699	4.20E-06	4.39E-07	2.00E-06
2.0	0.1	0.770284	0.770277	0.770272	0.770286	7.21E-06	1.18E-05	2.00E-06
	0.5	0.726464	0.726456	0.726449	0.726469	7.35E-06	1.49E-05	5.00E-06
	0.9	0.679109	0.679101	0.679095	0.679110	8.03E-06	1.43E-05	1.00E-06

**Table 12:** Numerical solutions of generalized Burgers′ equation by the proposed scheme and comparison with exact solutions, ADM [11], and CBRBF[13] for, *α* = 1, *β* = 0, and *δ* = 3.

		Exact	Proposed	ADM	CBRBF	Absolute errors		
*t*	*x*	*u_exact_*	$\overset{⌢}{u}\left(\eta \right)$	[11]	[13]	Proposed	ADM	CBRBF
0.0001	0.1	0.783660	0.783659	0.784106	-	4.55E-07	4.46E-04	-
	0.5	0.741285	0.741285	0.743145	-	5.66E-07	1.86E-03	-
	0.9	0.696157	0.696158	0.697089	-	7.00E-07	9.32E-04	-
0.0005	0.1	0.783670	0.783670	0.784115	0.783664	4.57E-07	4.45E-04	6.00E-06
	0.5	0.741296	0.741296	0.743150	0.741291	5.63E-07	1.85E-03	5.00E-06
	0.9	0.696169	0.696170	0.697089	0.696165	6.98E-07	9.20E-04	4.00E-06
0.001	0.1	0.783683	0.783682	0.784127	0.783664	4.60E-07	4.44E-04	1.90E-05
	0.5	0.741309	0.741309	0.743157	0.741293	5.61E-07	1.85E-03	1.60E-05
	0.9	0.696183	0.696184	0.697088	0.696168	6.95E-07	9.05E-04	1.50E-05

**Table 13:** Numerical solutions of generalized Burgers′ equation by the proposed scheme and comparison with exact solutions, and CBRBF [13] for *α* = 1, *β* = 0, and *δ* = 3.

		Exact	Proposed	CBRBF	Absolute errors	
*t*	*x*	*u_exact_*	$\overset{⌢}{u}\left(\eta \right)$	[13]	Proposed	CBRBF
0.5	0.1	0.796173	0.796174	0.796176	1.00E-06	3.00E-06
	0.5	0.75487	0.754871	0.754877	1.00E-06	7.00E-06
	0.9	0.710485	0.710486	0.710486	1.00E-06	1.00E-06
1.0	0.1	0.808297	0.808299	0.808299	2.00E-06	2.00E-06
	0.5	0.768157	0.768159	0.768165	2.00E-06	8.00E-06
	0.9	0.724622	0.724625	0.724623	3.00E-06	1.00E-06
2.0	0.1	0.831283	0.831288	0.831286	5.00E-06	3.00E-06
	0.5	0.793701	0.793706	0.793709	5.00E-06	8.00E-06
	0.9	0.752176	0.752182	0.752177	6.00E-06	1.00E-06
5.0	0.1	0.889248	0.88926	0.889252	1.20E-05	4.00E-06
	0.5	0.860439	0.860452	0.860452	1.30E-05	1.30E-05
	0.9	0.826825	0.826839	0.826828	1.40E-05	3.00E-06

Finally, we study the effect of change in the values of *c* and *d* in Equation (9) on the accuracy of approximate solution, and show the reliability of the proposed scheme. We used following test cases

Case (i) *p = c = 1 q = d = 1*

Case (ii) *p = c = 2 q = d = 2*

Case (iii) *p = c = 3 q = d = 3*

Case (iv) *p = c = 1 q = d = 2*

We consider the generalized Burgers′-Fisher Equation (1) with *α* = *β* = 0.001, and *δ* = 1. The approximate solution is obtained in the domain *x* ∈(0,1) and *t* ∈(0,1). GA has been used with the same settings for all the four cases (i)—(iv) as prescribed in Table 1 for example 1, except with a change in chromosome size for each case which is 8, 12, 16, and 10 for case(i), case(ii), case (ii), and case(iv) respectively. The approximate solutions have been obtained for each case and absolute errors have been computed. In Table 14 we provide the approximate solution obtained by the proposed scheme for each case at time *t = 0*.*1*. Table 15 shows average absolute errors obtained by the proposed scheme for each case (i)—(iv) for *t* ∈(0,1), also computational time and number of generations utilized are given for the sake of comparison. From the comparison of Table 15, it is observed that the average absolute error corresponding to case(i) with *p = c = 1* and *d = q = 1* is relatively very high compared to other cases (ii)–(iv). It is also observed that the accuracy is fairly equal for the remaining cases (ii)—(iv), however the computational time is quite different. It can be seen from Table 14 that for case (iv) we get the average absolute error fairly comparable to cases (ii) and (iii), but with lesser number generations and smaller computational time. Therefore it can be concluded on the basis of the simulation results that the choice of *c*, *d* have influence on the accuracy of approximate solutions and computational time. Nonetheless the comparison clearly demonstrates the accuracy and reliability of the proposed scheme.

**Table 14:** Comparison of approximate solutions with different values of *c* and *d* at *t = 0*.*1*.

		Proposed Scheme, $\overset{⌢}{u}\left(\eta \right)$			
	Exact	Case (i)	Case (ii)	Case (iii)	Case (iv)
*x*	*u_exact_*	*p = c = 1 q = d = 1*	*p = c = 2 q = d = 2*	*p = c = 3 q = d = 3*	*p = c = 1 q = d = 2*
0	0.500025	0.499641	0.500025	0.500025	0.500025
0.1	0.500013	0.499629	0.500012	0.500013	0.500013
0.2	0.500000	0.499616	0.500000	0.500000	0.500000
0.3	0.499988	0.499604	0.499988	0.499988	0.499988
0.4	0.499975	0.499591	0.499975	0.499975	0.499975
0.5	0.499963	0.499579	0.499963	0.499962	0.499963
0.6	0.499950	0.499566	0.499950	0.499950	0.499950
0.7	0.499938	0.499554	0.499938	0.499937	0.499938
0.8	0.499925	0.499541	0.499925	0.499925	0.499925
0.9	0.499913	0.499529	0.499913	0.499912	0.499913
1	0.499900	0.499516	0.499900	0.499900	0.499900

**Table 15:** Effect of change in *c* and *d* on the accuracy and computational time of the proposed scheme.

Values of *p*, *q*, *c*, *d*	Average absolute error	No. of generations	Computational time in sec
**Case (i): *p = c = 1q = d = 1***	1.91E-03	196	80
**Case (ii): *p = c = 2 q = d = 2***	1.97E-07	457	177
**Case (iii): *p = c = 3 q = d = 3***	1.42E-07	279	97
**Case (iv): *p = c = 1 q = d = 2***	1.76E-07	51	40

## Conclusions

A simple straightforward heuristic scheme based on the hybridization of Exp-function method and evolutionary algorithm has been proposed for obtaining the numerical solution of NPDEs. The proposed scheme has been successfully implemented for obtaining the numerical solutions of the generalized Burgers′-Fisher and Burgers′ equations. From the comparisons of numerical solutions made with the exact solutions, and some traditional methods including ADM, HPM, OHAM, and CBRBF, it can be concluded that the proposed scheme is effective and viable for solving such problems. Moreover, the beauty of the proposed scheme is that it can provide the approximate solution of the given NPDE on continuous values of time in the solution domain, once the unknown parameters are achieved.
